# The rising trend of emotional bullying in an Eastern Chinese City: a five-year epidemiological study among school-aged children (2020–2024)

**DOI:** 10.3389/fpubh.2025.1715737

**Published:** 2025-11-06

**Authors:** Liu Yang, Yuhan Li, Feifei Yan, Jianzhuo Li

**Affiliations:** Institute of School Health Surveillance, Jinan Center for Disease Control and Prevention, Jinan, China

**Keywords:** school bullying, physical bullying, emotional bullying, child, epidemiology, trend analysis, China

## Abstract

**Objective:**

To investigate the epidemiological characteristics and temporal trends of school bullying, with a particular emphasis on emotional forms, among students in Jinan, China, from 2020 to 2024.

**Methods:**

Data were collected from the Jinan Student Common Diseases and Health Influencing Factors Surveillance. A total of 84,289 participants were included through a stratified random cluster sampling design. The study assessed the prevalence and subtypes of school bullying, encompassing both physical and emotional bullying, and analyzed trends over 5 years.

**Results:**

The overall prevalence of school bullying significantly increased from 10.5% in 2020 to 14.6% in 2024 (*p* < 0.001). Notably, emotional bullying (14.4%) was markedly more prevalent than physical bullying (1.9%) in 2024. Significant disparities by sex were observed, with males reporting higher rates of total (16.4% vs. 12.7%), physical (2.4% vs. 1.4%), and emotional bullying (16.1% vs. 12.5%) compared to females (*p* < 0.001). While no significant urban-rural differences were found for total or emotional bullying, physical bullying was more common in urban areas (2.1% vs. 1.7%, *p* = 0.028). Primary schools exhibited the highest prevalence of total (17.4%) and physical bullying (2.8%). An analysis of emotional bullying subtypes revealed significant increases in teasing (from 9.4% to 13.3%), extortion (from 1.2% to 1.5%), and social exclusion (from 3.0% to 5.8%) from 2020 to 2024 (*p* < 0.001).

**Conclusion:**

School bullying, particularly emotional bullying, has risen significantly among students in Jinan. This increasing trend, especially in emotional bullying among younger students, underscores a critical public health issue that necessitates targeted intervention strategies. These findings provide valuable insights for developing evidence-based anti-bullying policies in Jinan and similar urban settings in China.

## 1 Introduction

School bullying represents a pervasive and significant public health challenge as well as a social issue that adversely impacts child development on a global scale. It is characterized by intentional, repetitive, aggressive behaviors occurring between individuals, marked by an imbalance of power within school environments or their surrounding contexts (1, 2). These behaviors encompass not only overt physical aggression but also more covert forms of emotional and psychological harm (3). The consequences for victims are profound and enduring, detrimentally affecting mental well-being during adolescence and often extending into adulthood. Long-term sequelae include psychological trauma, anxiety, depression, diminished self-esteem, and an elevated risk of suicidal ideation and behaviors (4). Furthermore, perpetrators of bullying are at a higher risk of engaging in violent behaviors, substance abuse, and criminal activities later in life, thereby perpetuating a damaging “cycle of harm” that poses a substantial threat to public health and social security (5–7).

Globally, the prevalence of school bullying exhibits significant regional variation. Data from the Global School-based Student Health Survey (GSHS) indicate that among adolescents aged 12 to 17 across 83 countries from 2003 to 2015, the pooled prevalence of those experiencing bullying at least once in the preceding 30 days was 30.5% (95% *CI*: 30.2–31.0%) (8). The highest rates were observed in the Eastern Mediterranean Region (45.1%) and the African Region (43.5%), while the lowest were reported in Europe (8.4%) (8). Evidence suggests that structured anti-bullying programs can significantly reduce both bullying perpetration (by 19%–20%) and victimization (by 15%–16%) (9). Nevertheless, in many countries and regions, factors such as insufficient public awareness, inadequate monitoring systems, and limited intervention resources contribute to underreporting and a lack of attention, implying that the true prevalence may substantially exceed currently published figures.

In China, rapid urbanization, evolving social structures, and transformations in family education patterns, coupled with the widespread proliferation of the internet have introduced new complexities to the prevention and control of school bullying (10, 11). Meta-analyses estimate that the prevalence of bullying victimization among Chinese school-aged populations ranges from 17.7% to 28.6%. However, the lack of consistent and standardized monitoring indicators, along with long-term dynamic data, hampers a precise understanding of the national landscape (12). A 2016 cross-sectional study conducted across seven provincial capitals reported rates of victimization, perpetration, and witnessing at 26.10%, 9.03%, and 28.90%, respectively (13). Furthermore, another study involving 95,873 students from 85 vocational schools found a victimization prevalence of 30.4% (14). A critical limitation of the existing literature is its predominant reliance on cross-sectional designs, which are often confined to single regions or specific educational stages. This approach fails to provide continuous dynamic surveillance of the same population over time, complicating the accurate delineation of long-term trends, the identification of high-risk groups, and the key influencing factors. Consequently, it hinders the formulation of targeted, evidence-based intervention strategies, particularly concerning emotional bullying in second-tier Chinese cities such as Jinan.

In this study, we utilized data from the 2024 Jinan Student Common Diseases and Health Influencing Factors Surveillance, integrated with historical data from 2020 to 2023, to conduct a comprehensive analysis. Our objective is to elucidate the temporal trends and epidemiological characteristics of school bullying among students in Jinan. The findings are expected to provide a scientific foundation for the local government to develop targeted anti-bullying policies and intervention programs, while also serving as a valuable reference for similar studies conducted in other Chinese cities.

## 2 Methods

### 2.1 Data source

This study is based on the Student Common Diseases and Health-Influencing Factors Monitoring Project. Surveys were conducted among students in grades 4 to 12 in Jinan from September to October each year. The 12 districts were categorized into six urban and six rural areas based on urbanization rates and economic levels. A stratified random cluster sampling method was employed according to the monitoring plan, whereby seven schools were randomly selected from each urban area (two primary schools, two junior high schools, two senior high schools, and one vocational high school) and five schools from each rural area (two primary schools, two junior high schools, and one senior high school). Stratification was performed by grade, and all students from two randomly selected classes within each grade were surveyed. The monitored schools remained consistent each year. In 2020, surveys were conducted in three urban and three rural areas; from 2021 to 2024, surveys were conducted across all 12 districts (Supplementary Figure S1). The number of participants from 2020 to 2024 was 8,510, 17,332, 18,681, 19,826, and 19,940, respectively. The data collected in this study are anonymous and do not involve personal privacy; therefore, informed consent is not required.

The survey was conducted in accordance with the unified requirements established by trained professionals, with quality control of the survey process overseen by supervisors.

### 2.2 Analysis indicators

This study investigates whether students have experienced (1) “malicious teasing or teasing due to physical defects or appearance,” (2) “extortion of money or property,” (3) “intentional exclusion from group activities or isolation,” (4) “threats or intimidation,” or (5) “physical aggression such as being hit, kicked, pushed, or shoved” on campus or in the surrounding areas within the past 30 days. Students who responded “sometimes” or “often” to any of the items (1)–(5) are classified as victims of bullying. Among these, those who indicated “sometimes” or “often” for any of the items (1)–(4) are defined as victims of emotional bullying, while students who responded “sometimes” or “often” to item (5) are classified as victims of physical bullying.

### 2.3 Statistical analysis

Data entry was conducted using EpiData software, and statistical analyses were performed using R version 4.4.1. Categorical variables were expressed as frequencies and percentages (composition ratios). The *Chi-square* test was employed to analyze differences in the occurrence of various types of bullying based on sex, district, and school type. Additionally, the *Chi-square* trend test was utilized to examine temporal trends in the occurrence of different types of bullying. To address potential confounding due to demographic differences across years, a multivariate logistic regression model was employed, adjusting for sex, district, type of school, and survey year as a continuous variable. Sensitivity analyses were conducted to assess the comparability of the 2020 sample with samples from 2021 to 2024. A two-sided test was performed with a significance level of *α* = 0.05.

## 3 Results

### 3.1 Epidemiological characteristics of school bullying among children in 2024

In 2024, a total of 19,940 children were surveyed, revealing an overall prevalence of school bullying at 14.6% (2,914/19,940). Among these, the prevalence of physical bullying was 1.9% (384/19,940), while emotional bullying was reported at 14.4% (2,864/19,940). Significant differences were observed in the prevalence of total school bullying, physical bullying, and emotional bullying based on sex (*p* < 0.001 for all comparisons). Specifically, the prevalence of total school bullying was higher among males (16.4%) compared to females (12.7%). Likewise, the prevalence of physical bullying was 2.4% in males versus 1.4% in females, and the prevalence of emotional bullying was 16.1% in males compared to 12.5% in females. When analyzing by district, no significant differences were found in the prevalence of total school bullying (*p* = 0.959) or emotional bullying (*p* = 0.950) between urban and rural areas. However, a significant difference was observed in the prevalence of physical bullying (*p* = 0.028), with urban areas exhibiting a higher prevalence (2.1%) compared to rural areas (1.7%). Regarding school type, significant differences were evident in the prevalence of total school bullying, physical bullying, and emotional bullying (*p* < 0.001 for all comparisons). Primary schools reported the highest prevalence of total school bullying (17.4%) and physical bullying (2.8%), followed by junior high schools (15.5% for total bullying and 1.6% for physical bullying). In contrast, senior high schools had the lowest prevalence of total school bullying (9.8%) and emotional bullying (9.7%). The prevalence of total school bullying in vocational high schools was 12.8%, with a physical bullying prevalence of 1.7% (Table 1).

**Table 1:** Epidemiological characteristics of school bullying among children in 2024.

Variables	Number of respondents, (*n*, %)	Bullying		Physical bullying		Emotional bullying	
		Yes (*n*, %)	*p*	Yes (*n*, %)	*p*	Yes (*n*, %)	*p*
Total	19,940 (100)	2,914 (14.6)	—	384 (1.9)	—	2,864 (14.4)	—
Sex			<0.001		<0.001		<0.001
Male	10,305 (51.7)	1,690 (16.4)		251 (2.4)		1,660 (16.1)	
Female	9,635 (48.3)	1,224 (12.7)		133 (1.4)		1,204 (12.5)	
District			0.959		0.028		0.950
Urban	11,053 (55.4)	1,614 (14.6)		234 (2.1)		1,586 (14.3)	
Rural	8,887 (44.6)	1,300 (14.6)		150 (1.7)		1,278 (14.4)	
Type of school			<0.001		<0.001		<0.001
Primary school	6,877 (34.5)	1,194 (17.4)		195 (2.8)		1,163 (16.9)	
Junior high school	7,031 (35.3)	1,093 (15.5)		114 (1.6)		1,081 (15.4)	
Senior high school	4,869 (24.4)	478 (9.8)		55 (1.1)		473 (9.7)	
Vocational high school	1,163 (5.8)	149 (12.8)		20 (1.7)		147 (12.6)	

Percentages are calculated based on the total number of respondents in each category.

### 3.2 Epidemiological characteristics of emotional bullying among children in 2024

In 2024, the prevalence rates for various forms of emotional bullying were as follows: teasing (13.3%, 2,654/19,940), extortion (1.5%, 296/19,940), social exclusion (5.8%, 1,158/19,940), and threats (2.2%, 433/19,940). Significant sex differences were noted in the prevalence of teasing (*p* < 0.001), extortion (*p* < 0.001), and threats (*p* < 0.001). Males exhibited a higher prevalence of teasing (15.2% compared to 11.3% in females), extortion (1.8% vs. 1.2% in females), and threats (2.6% vs. 1.7% in females). Conversely, the prevalence of social exclusion did not differ significantly between males and females (*p* = 0.783). Regarding geographic location, no significant difference was observed in the prevalence of teasing between urban and rural areas (*p* = 0.353). However, significant differences were found in the prevalence of extortion (*p* = 0.002), social exclusion (*p* < 0.001), and threats (*p* = 0.019). Urban areas reported a higher prevalence of extortion (1.7% vs. 1.2% in rural areas), while rural areas exhibited a greater prevalence of social exclusion (6.9% vs. 4.9% in urban areas) and threats (2.4% vs. 1.9% in urban areas). In terms of school type, significant differences were observed in the prevalence of teasing (*p* < 0.001), extortion (*p* = 0.033), social exclusion (*p* < 0.001), and threats (*p* = 0.002). Primary schools exhibited the highest prevalence of teasing (15.4%) and social exclusion (8.5%). Junior high schools also showed a relatively high prevalence of teasing (14.6%). In contrast, senior high schools had the lowest prevalence of teasing (8.9%) and social exclusion (3.7%). Vocational high schools reported a higher prevalence of extortion (2.5%) and threats (2.5%) compared to other school types in certain instances (Table 2).

**Table 2:** Epidemiological characteristics of emotional bullying among children in 2024.

Variables	Number of respondents, *n* (%)	Teasing		Extortion		Social exclusion		Threats	
		*n* (%)	*p*	*n* (%)	*p*	*n* (%)	*p*	*n* (%)	*p*
Total	19,940 (100)	2,654 (13.3)	—	296 (1.5)	—	1,158 (5.8)	—	433 (2.2)	—
Sex			<0.001		<0.001		0.783		<0.001
Male	10,305 (51.7)	1,569 (15.2)		184 (1.8)		603 (5.9)		270 (2.6)	
Female	9,635 (48.3)	1,085 (11.3)		112 (1.2)		555 (5.8)		163 (1.7)	
District			0.353		0.002		<0.001		0.019
Urban	11,053 (55.4)	1,449 (13.1)		190 (1.7)		542 (4.9)		264 (2.4)	
Rural	8,887 (44.6)	1,205 (13.6)		106 (1.2)		616 (6.9)		169 (1.9)	
Type of school			<0.001		0.033		<0.001		0.002
Primary school	6,877 (34.5)	1,057 (15.4)		95 (1.4)		586 (8.5)		169 (2.5)	
Junior high school	7,031 (35.3)	1,026 (14.6)		103 (1.5)		337 (4.8)		163 (2.3)	
Senior high school	4,869 (24.4)	435 (8.9)		69 (1.4)		182 (3.7)		72 (1.5)	
Vocational high school	1,163 (5.8)	136 (11.7)		29 (2.5)		53 (4.6)		29 (2.5)	

Percentages are calculated based on the total number of respondents in each category.

### 3.3 Trends of school bullying from 2020 to 2024

**Figure 1:**
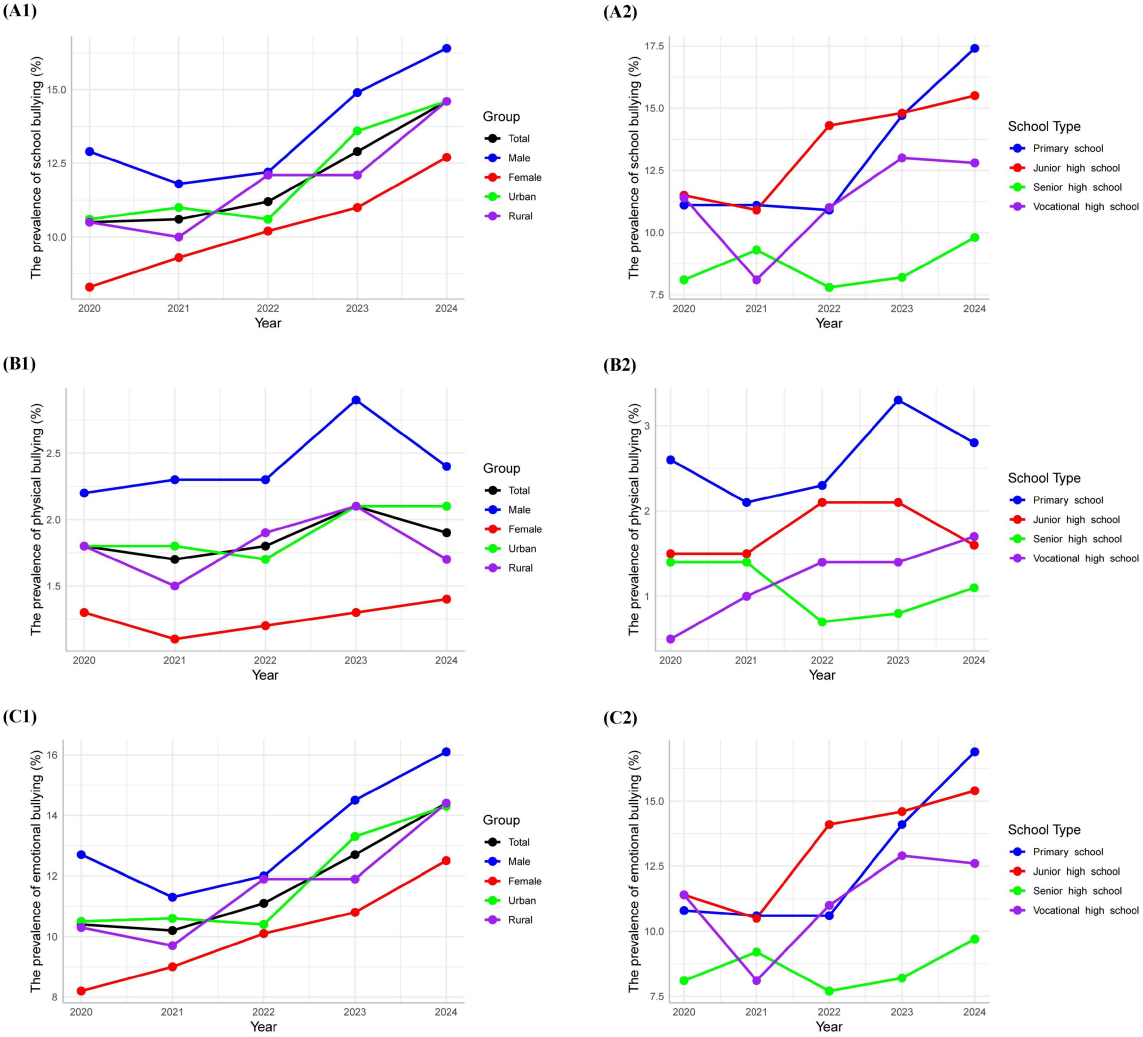
The trends of school bullying, physical bullying, and emotional bullying from 2020 to 2024.

The overall prevalence of school bullying exhibited a significant upward trend from 2020 to 2024 (*p* < 0.001), increasing from 10.5% in 2020 to 14.6% in 2024. Both male and female students experienced a significant rise in prevalence over the years (*p* < 0.001 for both). Specifically, the prevalence among males increased from 12.9% in 2020 to 16.4% in 2024, while for females, it rose from 8.3% to 12.7%. Urban and rural areas both demonstrated significant increases in the prevalence of total bullying (*p* < 0.001 for both), with urban areas rising from 10.6% to 14.6% and rural areas from 10.5% to 14.6%. Among different school types, primary schools, junior high schools, and vocational high schools exhibited significant upward trends in the prevalence of total bullying (*p* < 0.001 for all), whereas senior high schools did not show a significant trend (*p* = 0.110). The overall prevalence of physical bullying also demonstrated a significant trend from 2020 to 2024 (*p* = 0.017), starting at 1.8% in 2020, fluctuating to 1.7% in 2021, 1.8% in 2022, increasing to 2.1% in 2023, and finally settling at 1.9% in 2024. Males experienced a non-significant increasing trend (*p* = 0.081), rising from 2.3% in 2020 to 2.4% in 2024, while females demonstrated a significant increase (*p* = 0.190), from 1.2% to 1.4%. Urban areas revealed a significant increasing trend (*p* = 0.020), from 1.8% to 2.1%, whereas rural areas did not show a significant trend (*p* = 0.343). Primary schools had a significant increasing trend in the prevalence of physical bullying (*p* = 0.002), from 1.6% to 2.8%. Junior high schools, senior high schools, and vocational high schools did not exhibit significant trends (*p* = 0.236, 0.082, and 0.052 respectively). Finally, the overall prevalence of emotional bullying showed a significant increasing trend from 2020 to 2024 (*p* < 0.001), rising from 10.4% in 2020 to 14.4% in 2024. Both male and female students displayed significant increasing trends (*p* < 0.001 for both), with males increasing from 12.7% to 16.1% and females from 8.2% to 12.5%. Urban and rural areas also exhibited significant upward trends (*p* < 0.001 for both), with urban areas rising from 10.5% to 14.3% and rural areas from 10.3 to 14.4%. Among school types, primary schools and junior high schools showed significant increasing trends (*p* < 0.001 for both), while senior high schools did not show a significant trend (*p* = 0.117) and vocational high schools had a significant increasing trend (*p* = 0.031) (Figure 1; Supplementary Table S1).

The multivariate logistic regression models indicated that the increasing temporal trend remained statistically significant. The adjusted odds of bullying, physical bullying, and emotional bullying increased by 12%, 5%, and 12% per year, respectively (Supplementary Table S2).

### 3.4 Trends of specific emotional bullying from 2020 to 2024

**Figure 2:**
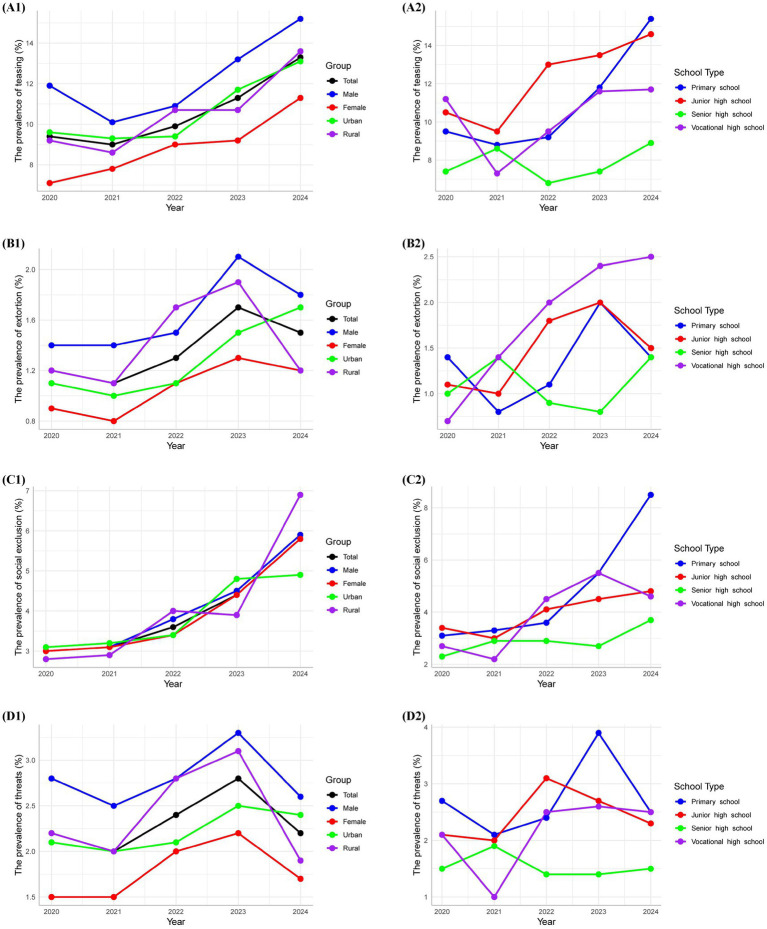
The trends of specific emotional bullying from 2020 to 2024.

The prevalence of teasing exhibited a significant increase, rising from 9.4% in 2020 to 13.3% in 2024 (*p* < 0.001). This trend was consistent across both sexes, with males increasing from 11.9% to 15.2% and females from 7.1% to 11.3%. Additionally, urban areas showed a rise from 9.6% to 13.1%, while rural areas increased from 9.2% to 13.6%. Notable uptrends in teasing were documented in primary, junior high, and vocational high schools (*p* < 0.001), whereas senior high schools did not demonstrate significant changes (*p* = 0.149). Regarding extortion, the overall prevalence rose significantly from 1.2% to 1.5% (*p* < 0.001). Both males (1.4% to 1.8%) and females (0.9% to 1.2%) experienced significant increases. Urban areas displayed a significant uptrend, increasing from 1.1% to 1.7% (*p* < 0.001), while rural areas did not show significant changes (*p* = 0.227). Significant increases were also observed in primary, junior high, and vocational high schools (*p* = 0.005, 0.002, 0.181), whereas senior high schools exhibited no significant change (*p* = 0.781). The prevalence of social exclusion rose significantly from 3.0% to 5.8% (*p* < 0.001). Both males (3.0% to 5.9%) and females (3.0% to 5.8%) in urban (2.8% to 4.9%) and rural (3.1% to 6.9%) areas experienced significant increases. Significant uptrends were noted in primary, junior high, and vocational high schools (*p* < 0.001), while senior high schools did not show significant changes (*p* = 0.011). In terms of threats, no significant overall trend was observed (*p* = 0.059, 2.0% to 2.8%). However, urban areas exhibited a significant increase from 2.1% to 2.4% (*p* = 0.025), while other demographics, including males, females, rural areas, and most school types, did not demonstrate significant trends (Figure 2; Supplementary Table S3).

The multivariate logistic regression models confirmed that the observed increasing temporal trend remained statistically significant. Specifically, the adjusted odds of teasing, extortion, and social exclusion increased by 13%, 10%, and 22% per year, respectively (Supplementary Table S2).

### 3.5 Sensitivity analysis

To evaluate the comparability of the 2020 sample, a sensitivity analysis was conducted by excluding the 2020 data. This analysis confirmed that the significant increasing trend persisted from 2021 to 2024, thereby reinforcing the robustness of our primary findings (Supplementary Tables S4, S5).

## 4 Discussion

This study presents a systematic epidemiological analysis of school bullying trends from 2020 to 2024 among students in grades 4 to 12 in Jinan, China. The key findings indicate a significant upward trend in the overall prevalence of bullying, distinct patterns across demographic subgroups, and a predominance of emotional bullying over physical bullying. In 2024, the overall prevalence of school bullying in Jinan was 14.6%, which is notably lower than the 53.5% reported in a national study (15). While regional socioeconomic differences, such as Jinan’s status as a provincial capital with potentially greater resources, may contribute to this discrepancy, the national study (15) employed a broader definition that encompasses not only the behaviors we investigated but also includes items such as deliberate damage to property, theft of belongings, and exclusion from classes. Furthermore, differences in study populations can substantially influence prevalence estimates (16). Therefore, direct numerical comparisons should be approached with caution. The value of our data lies not in the absolute prevalence figure but in the internally consistent trends and patterns observed over time using a standardized methodology, which reliably reflects the escalating challenge within Jinan. Consistent with global literature (17, 18), emotional bullying was significantly more prevalent than physical bullying, underscoring that the most common forms of victimization are often emotional and potentially less visible to adults.

Consistent with established research (19, 20), significant sex disparities were observed, with males reporting higher rates of total, physical, and emotional bullying compared to females. This pattern is frequently attributed to societal gender norms that may tolerate or even encourage overt aggression in boys, a phenomenon that is particularly pronounced within the context of Chinese masculine social expectations. Interestingly, social exclusion was the only form of emotional bullying that did not differ significantly by sex, suggesting that this behavior may be influenced more by universal peer group dynamics than by gendered social scripts (19, 21, 22). Contrary to earlier Chinese studies indicating higher bullying rates in rural areas—often linked to resource limitations and parental migration (23)—our analysis found no significant urban-rural difference in the prevalence of total or emotional bullying in 2024. This convergence may reflect Jinan’s rapid urbanization and development, which have narrowed disparities in school infrastructure and teacher training. However, the slightly higher rate of physical bullying in urban areas (2.1% vs. 1.7%) warrants further investigation; potential contributors could include higher population density, increased academic competition, and greater exposure to aggressive media content. The analysis by school type followed a recognizable developmental pattern: primary schools exhibited the highest rates of total and physical bullying, which decreased progressively through junior high and senior high schools. This trend aligns with developmental psychology theories positing that younger children have less mature emotional regulation and conflict-resolution skills, often resorting to physicality, while adolescents develop more sophisticated—though sometimes equally harmful—social strategies (24, 25). Vocational high schools presented a distinct profile, with a moderate overall bullying rate but relatively higher instances of extortion and threats. This may be linked to the unique pressures and social environments characterizing vocational education pathways.

The most concerning finding is the significant upward trend in bullying prevalence, which increased from 10.5% in 2020 to 14.6% in 2024. This trend sharply contrasts with the declining rates observed in many developed countries during the same period, despite similar pandemic conditions (26). This discrepancy suggests that the universal disruptions caused by COVID-19 alone are insufficient to account for this phenomenon. We propose that the pandemic acted as a catalyst, exacerbating pre-existing, locality-specific socio-educational factors within Jinan. Prolonged isolation, disrupted routines, and heightened family stress during lockdowns may have compromised socio-emotional skills and increased aggression upon the return to in-person schooling (27, 28). However, the persistence and growth of these effects beyond the acute phase of the pandemic underscore deeper systemic issues that interact with the aftermath of the pandemic. There are two key factors specific to the context of a highly competitive urban center in China, such as Jinan, that elucidate this divergent trend. First, the immense academic pressure associated with high-stakes examination systems (e.g., the Zhongkao and Gaokao) is a well-documented stressor (29). The post-lockdown period likely intensified the focus on academic remediation and the necessity to “catch up” on lost curriculum time. This heightened pressure-cooker environment may have escalated student anxiety and frustration, manifesting as interpersonal aggression and eroding empathy, thereby creating a fertile ground for bullying (30). Concurrently, the overwhelming emphasis on academic achievement may have diverted school resources and attention away from robust social-emotional learning (SEL) and proactive bullying monitoring, effectively fostering a permissive environment for peer victimization. Second, the social dynamics among children in urban Chinese families, many of whom are only children, may also contribute to this phenomenon. The absence of siblings can sometimes limit opportunities for developing conflict-resolution and empathy skills within the family unit (31). The pandemic-enforced social isolation critically disrupted the vital peer-to-peer interactions necessary for practicing these skills. Consequently, upon returning to school, some students may have lacked the mature social tools to navigate complex hierarchies, resorting to bullying behaviors as a maladaptive coping mechanism.

The observation that senior high schools are the only group not exhibiting a significant upward trend supports the notion of greater emotional maturity, a more stable social environment, and an increased awareness of serious consequences among senior high school students. Furthermore, older students may possess more developed critical thinking skills, enabling them to navigate online environments effectively. However, the widespread rise of social media and digital platforms may have normalized aggressive online behaviors that can spill over into school settings (32, 33). In contrast, younger students in primary schools are particularly vulnerable. They not only face the academic and social pressures mentioned earlier but are also developmentally less equipped to regulate emotions or resolve conflicts constructively. This situation highlights the sharp increase in physical bullying and underscores the urgent need for evidence-based social-emotional learning interventions at this critical developmental stage.

Among the emotional bullying subtypes, teasing remains the most prevalent form, while social exclusion has exhibited the most significant relative increase throughout the study period. The high prevalence of teasing is particularly insidious, as it is often dismissed as “harmless,” despite its strong associations with anxiety, depression, and low self-esteem (34, 35). The substantial rise in social exclusion—a behavior that fundamentally undermines a child’s sense of belonging and identity—is equally concerning and may represent a specific manifestation of post-pandemic social fragmentation, potentially linked to long-term social withdrawal and psychological distress (36, 37). Furthermore, the regional variations in subtypes, including the higher incidence of extortion in urban areas and the increased prevalence of exclusion and threats in rural areas, suggest that anti-bullying policies cannot adopt a one-size-fits-all approach; rather, they must be tailored to effectively address these distinct local manifestations and underlying social dynamics.

This study has several limitations. Firstly, the reliance on self-reported data is susceptible to both recall bias and social desirability bias. Future research would benefit from adopting a multi-informant approach that incorporates reports from both teachers and parents. Secondly, this study focused exclusively on victimization experiences and did not collect data on the roles of perpetrators or bystanders, which limits a comprehensive understanding of the bully-victim dynamic and the overall social ecology of bullying. Thirdly, the scope was restricted to traditional bullying (e.g., physical and emotional bullying); by not including cyberbullying—an increasingly prevalent form of peer harm manifested through social media platforms, instant messaging apps, and online forums—this study overlooks a crucial and interconnected aspect of contemporary peer victimization that likely interacts with the observed trends. Future studies could further explore the specific role of cyberbullying (e.g., how it co-occurs with traditional bullying or exerts independent impacts on victims) to enrich the understanding of bullying’s modern landscape. Lastly, since the study was conducted solely in Jinan, the generalizability of the findings to other Chinese cities with diverse socio-economic contexts may be limited.

## 5 Conclusion

This five-year trend analysis (2020–2024) reveals a significant and concerning increase in the prevalence of school bullying among students in Jinan, China, primarily driven by a rise in emotional victimization. Notably, males experience higher rates of total, physical, and emotional bullying compared to females, while urban areas exhibit a greater prevalence of physical bullying than rural areas. Primary schools report the highest rates of total and physical bullying, indicating that younger students may require targeted interventions. This study underscores the necessity for effective anti-bullying policies and interventions, particularly in light of the increasing trends observed. Future longitudinal research should incorporate data on perpetrators and metrics related to cyberbullying to provide a more comprehensive understanding of the bullying ecosystem, thereby informing more effective and holistic prevention strategies, with a particular focus on social-emotional learning for younger students and context-specific interventions for vocational schools.

## Data Availability

The data supporting the findings of this study are available from the corresponding author upon reasonable request. Requests to access the datasets should be directed to LY, yangliu_1987@163.com.
