# Supplementary material for: The rising trend of emotional bullying in an Eastern Chinese City: a five-year epidemiological study among school-aged children (2020–2024)
Source: Front Public Health. 2025 Nov 6;13:1715737. doi: 10.3389/fpubh.2025.1715737 (PMC12630124; doi:10.3389/fpubh.2025.1715737)
Supplement: Supplementary file 1 [file Data_Sheet_1.pdf]

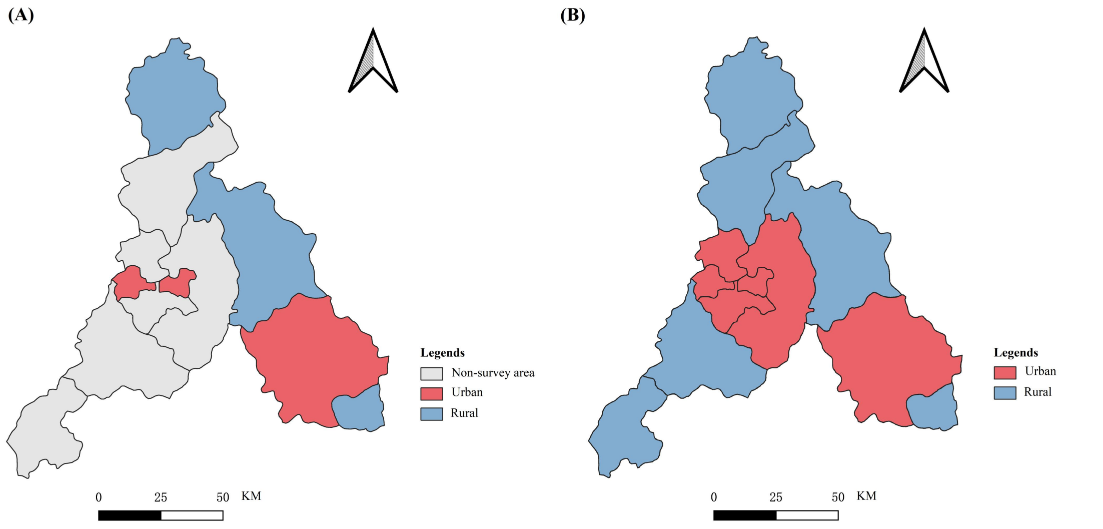

Figure S1. The district distribution of survey. (A) survey areas for 2020; (B) survey areas for 2021-2024 (the map was generated using QGIS 3.30.0, https://qgis.org/).

Table S1. The trends of school bullying from 2020 to 2024.

	2020	2021	2022	2023	2024	*Trend-P*
**Bullying, n (%)**						
Total	897 (10.5)	1829 (10.6)	2100 (11.2)	2567 (12.9)	2914 (14.6)	< 0.001
Male	536 (12.9)	1031 (11.8)	1171 (12.2)	1504 (14.9)	1690 (16.4)	< 0.001
Female	361 (8.3)	798 (9.3)	929 (10.2)	1063 (11.0)	1224 (12.7)	< 0.001
Urban	520 (10.6)	1024 (11.0)	1116 (10.6)	1493 (13.6)	1614 (14.6)	< 0.001
Rural	377 (10.5)	805 (10.0)	984 (12.1)	1074 (12.1)	1300 (14.6)	< 0.001
Primary school	324 (11.1)	703 (11.1)	658 (10.9)	978 (14.7)	1194 (17.4)	< 0.001
Junior high school	350 (11.5)	714 (10.9)	928 (14.3)	1005 (14.8)	1093 (15.5)	< 0.001
Senior high school	173 (8.1)	372 (9.3)	389 (7.8)	419 (8.2)	478 (9.8)	0.110
Vocational high school	50 (11.4)	40 (8.1)	125 (11.0)	165 (13.0)	149 (12.8)	0.023
**Physical bullying, n (%)**						
Total	151 (1.8)	293 (1.7)	327 (1.8)	424 (2.1)	384 (1.9)	0.017
Male	93 (2.2)	201 (2.3)	218 (2.3)	298 (2.9)	251 (2.4)	0.081
Female	58 (1.3)	92 (1.1)	109 (1.2)	126 (1.3)	133 (1.4)	0.190
Urban	86 (1.8)	171 (1.8)	175 (1.7)	235 (2.1)	234 (2.1)	0.020
Rural	65 (1.8)	122 (1.5)	152 (1.9)	189 (2.1)	150 (1.7)	0.343
Primary school	75 (2.6)	132 (2.1)	138 (2.3)	219 (3.3)	195 (2.8)	0.002
Junior high school	44 (1.5)	100 (1.5)	138 (2.1)	146 (2.1)	114 (1.6)	0.236
Senior high school	30 (1.4)	56 (1.4)	35 (0.7)	41 (0.8)	55 (1.1)	0.082
Vocational high school	2 (0.5)	5 (1.0)	16 (1.4)	18 (1.4)	20 (1.7)	0.052
**Emotional bullying, n (%)**						
Total	883 (10.4)	1765 (10.2)	2068 (11.1)	2516 (12.7)	2864 (14.4)	< 0.001
Male	527 (12.7)	988 (11.3)	1148 (12.0)	1468 (14.5)	1660 (16.1)	< 0.001
Female	356 (8.2)	777 (9.0)	920 (10.1)	1048 (10.8)	1204 (12.5)	< 0.001
Urban	513 (10.5)	985 (10.6)	1097 (10.4)	1459 (13.3)	1586 (14.3)	< 0.001
Rural	370 (10.3)	780 (9.7)	971 (11.9)	1057 (11.9)	1278 (14.4)	< 0.001
Primary school	314 (10.8)	670 (10.6)	641 (10.6)	941 (14.1)	1163 (16.9)	< 0.001
Junior high school	347 (11.4)	687 (10.5)	916 (14.1)	995 (14.6)	1081 (15.4)	< 0.001
Senior high school	172 (8.1)	368 (9.2)	386 (7.7)	416 (8.2)	473 (9.7)	0.117
Vocational high school	50 (11.4)	40 (8.1)	125 (11.0)	164 (12.9)	147 (12.6)	0.031

Table S2. The effect of year on bullying by multivariate logistic regression models.

	OR	**95% *CI***	***P***
Bullying	1.12	1.10, 1.14	< 0.001
Physical bullying	1.05	1.01, 1.09	0.019
Emotional bullying	1.12	1.10, 1.14	< 0.001
Teasing	1.13	1.11, 1.15	< 0.001
Extortion	1.10	1.05, 1.15	< 0.001
Social exclusion	1.22	1.19, 1.26	< 0.001
Threats	1.03	1.00, 1.07	0.077

Note: adjusted for sex (male/female), district (urban/rural), type of school (primary school/junior high school/senior high school/vocational high school), and survey year as a continuous variable.

Table S3. The trends of specific emotional bullying from 2020 to 2024.

	2020	2021	2022	2023	2024	*Trend-P*
**Teasing, n (%)**						
Total	802 (9.4)	1556 (9.0)	1856 (9.9)	2232 (11.3)	2654 (13.3)	< 0.001
Male	493 (11.9)	881 (10.1)	1041 (10.9)	1337 (13.2)	1569 (15.2)	< 0.001
Female	309 (7.1)	675 (7.8)	815 (9.0)	895 (9.2)	1085 (11.3)	< 0.001
Urban	472 (9.6)	863 (9.3)	984 (9.4)	1285 (11.7)	1449 (13.1)	< 0.001
Rural	330 (9.2)	693 (8.6)	872 (10.7)	947 (10.7)	1205 (13.6)	< 0.001
Primary school	276 (9.5)	558 (8.8)	560 (9.2)	787 (11.8)	1057 (15.4)	< 0.001
Junior high school	320 (10.5)	618 (9.5)	846 (13.0)	919 (13.5)	1026 (14.6)	< 0.001
Senior high school	157 (7.4)	344 (8.6)	342 (6.8)	379 (7.4)	435 (8.9)	0.149
Vocational high school	49 (11.2)	36 (7.3)	108 (9.5)	147 (11.6)	136 (11.7)	0.054
**Extortion, n (%)**						
Total	99 (1.2)	184 (1.1)	249 (1.3)	337 (1.7)	296 (1.5)	< 0.001
Male	58 (1.4)	119 (1.4)	145 (1.5)	209 (2.1)	184 (1.8)	0.001
Female	41 (0.9)	65 (0.8)	104 (1.1)	128 (1.3)	112 (1.2)	0.004
Urban	54 (1.1)	96 (1.0)	111 (1.1)	170 (1.5)	190 (1.7)	< 0.001
Rural	45 (1.2)	88 (1.1)	138 (1.7)	167 (1.9)	106 (1.2)	0.227
Primary school	40 (1.4)	52 (0.8)	69 (1.1)	132 (2.0)	95 (1.4)	0.002
Junior high school	34 (1.1)	68 (1.0)	114 (1.8)	134 (2.0)	103 (1.5)	0.005
Senior high school	22 (1.0)	57 (1.4)	43 (0.9)	41 (0.8)	69 (1.4)	0.781
Vocational high school	3 (0.7)	7 (1.4)	23 (2.0)	30 (2.4)	29 (2.5)	0.013
**Social exclusion, n (%)**						
Total	254 (3.0)	531 (3.1)	680 (3.6)	876 (4.4)	1158 (5.8)	< 0.001
Male	124 (3.0)	266 (3.1)	369 (3.8)	453 (4.5)	603 (5.9)	< 0.001
Female	130 (3.0)	265 (3.1)	311 (3.4)	423 (4.4)	555 (5.8)	< 0.001
Urban	152 (3.1)	295 (3.2)	354 (3.4)	527 (4.8)	542 (4.9)	< 0.001
Rural	102 (2.8)	236 (2.9)	326 (4.0)	349 (3.9)	616 (6.9)	< 0.001
Primary school	91 (3.1)	208 (3.3)	219 (3.6)	364 (5.5)	586 (8.5)	< 0.001
Junior high school	103 (3.4)	195 (3.0)	266 (4.1)	303 (4.5)	337 (4.8)	< 0.001
Senior high school	48 (2.3)	117 (2.9)	144 (2.9)	139 (2.7)	182 (3.7)	0.004
Vocational high school	12 (2.7)	11 (2.2)	51 (4.5)	70 (5.5)	53 (4.6)	0.011
**Threats, n (%)**						
Total	183 (2.2)	349 (2.0)	448 (2.4)	546 (2.8)	433 (2.2)	0.059
Male	117 (2.8)	221 (2.5)	265 (2.8)	337 (3.3)	270 (2.6)	0.444
Female	66 (1.5)	128 (1.5)	183 (2.0)	209 (2.2)	163 (1.7)	0.067
Urban	102 (2.1)	189 (2.0)	218 (2.1)	271 (2.5)	264 (2.4)	0.025
Rural	81 (2.2)	160 (2.0)	230 (2.8)	275 (3.1)	169 (1.9)	0.717
Primary school	78 (2.7)	134 (2.1)	146 (2.4)	257 (3.9)	169 (2.5)	0.027
Junior high school	64 (2.1)	133 (2.0)	201 (3.1)	187 (2.7)	163 (2.3)	0.206
Senior high school	32 (1.5)	77 (1.9)	72 (1.4)	69 (1.4)	72 (1.5)	0.211
Vocational high school	9 (2.1)	5 (1.0)	29 (2.5)	33 (2.6)	29 (2.5)	0.204

Table S4. Sensitivity analysis of the trends of school bullying by excluding 2020.

	2021	2022	2023	2024	*Trend-P*
**Bullying, n (%)**					
Total	1829 (10.6)	2100 (11.2)	2567 (12.9)	2914 (14.6)	< 0.001
Male	1031 (11.8)	1171 (12.2)	1504 (14.9)	1690 (16.4)	< 0.001
Female	798 (9.3)	929 (10.2)	1063 (11.0)	1224 (12.7)	< 0.001
Urban	1024 (11.0)	1116 (10.6)	1493 (13.6)	1614 (14.6)	< 0.001
Rural	805 (10.0)	984 (12.1)	1074 (12.1)	1300 (14.6)	< 0.001
Primary school	703 (11.1)	658 (10.9)	978 (14.7)	1194 (17.4)	< 0.001
Junior high school	714 (10.9)	928 (14.3)	1005 (14.8)	1093 (15.5)	< 0.001
Senior high school	372 (9.3)	389 (7.8)	419 (8.2)	478 (9.8)	0.194
Vocational high school	40 (8.1)	125 (11.0)	165 (13.0)	149 (12.8)	0.005
**Physical bullying, n (%)**					
Total	293 (1.7)	327 (1.8)	424 (2.1)	384 (1.9)	0.015
Male	201 (2.3)	218 (2.3)	298 (2.9)	251 (2.4)	0.154
Female	92 (1.1)	109 (1.2)	126 (1.3)	133 (1.4)	0.045
Urban	171 (1.8)	175 (1.7)	235 (2.1)	234 (2.1)	0.029
Rural	122 (1.5)	152 (1.9)	189 (2.1)	150 (1.7)	0.245
Primary school	132 (2.1)	138 (2.3)	219 (3.3)	195 (2.8)	< 0.001
Junior high school	100 (1.5)	138 (2.1)	146 (2.1)	114 (1.6)	0.751
Senior high school	56 (1.4)	35 (0.7)	41 (0.8)	55 (1.1)	0.419
Vocational high school	5 (1.0)	16 (1.4)	18 (1.4)	20 (1.7)	0.291
**Emotional bullying, n (%)**					
Total	1765 (10.2)	2068 (11.1)	2516 (12.7)	2864 (14.4)	< 0.001
Male	988 (11.3)	1148 (12.0)	1468 (14.5)	1660 (16.1)	< 0.001
Female	777 (9.0)	920 (10.1)	1048 (10.8)	1204 (12.5)	< 0.001
Urban	985 (10.6)	1097 (10.4)	1459 (13.3)	1586 (14.3)	< 0.001
Rural	780 (9.7)	971 (11.9)	1057 (11.9)	1278 (14.4)	< 0.001
Primary school	670 (10.6)	641 (10.6)	941 (14.1)	1163 (16.9)	< 0.001
Junior high school	687 (10.5)	916 (14.1)	995 (14.6)	1081 (15.4)	< 0.001
Senior high school	368 (9.2)	386 (7.7)	416 (8.2)	473 (9.7)	0.196
Vocational high school	40 (8.1)	125 (11.0)	164 (12.9)	147 (12.6)	0.007

Table S5. Sensitivity analysis of the trends of specific emotional bullying by excluding 2020.

	2021	2022	2023	2024	*Trend-P*
**Teasing, n (%)**					
Total	1556 (9.0)	1856 (9.9)	2232 (11.3)	2654 (13.3)	< 0.001
Male	881 (10.1)	1041 (10.9)	1337 (13.2)	1569 (15.2)	< 0.001
Female	675 (7.8)	815 (9.0)	895 (9.2)	1085 (11.3)	< 0.001
Urban	863 (9.3)	984 (9.4)	1285 (11.7)	1449 (13.1)	< 0.001
Rural	693 (8.6)	872 (10.7)	947 (10.7)	1205 (13.6)	< 0.001
Primary school	558 (8.8)	560 (9.2)	787 (11.8)	1057 (15.4)	< 0.001
Junior high school	618 (9.5)	846 (13.0)	919 (13.5)	1026 (14.6)	< 0.001
Senior high school	344 (8.6)	342 (6.8)	379 (7.4)	435 (8.9)	0.242
Vocational high school	36 (7.3)	108 (9.5)	147 (11.6)	136 (11.7)	0.003
**Extortion, n (%)**					
Total	184 (1.1)	249 (1.3)	337 (1.7)	296 (1.5)	< 0.001
Male	119 (1.4)	145 (1.5)	209 (2.1)	184 (1.8)	0.003
Female	65 (0.8)	104 (1.1)	128 (1.3)	112 (1.2)	0.005
Urban	96 (1.0)	111 (1.1)	170 (1.5)	190 (1.7)	< 0.001
Rural	88 (1.1)	138 (1.7)	167 (1.9)	106 (1.2)	0.463
Primary school	52 (0.8)	69 (1.1)	132 (2.0)	95 (1.4)	< 0.001
Junior high school	68 (1.0)	114 (1.8)	134 (2.0)	103 (1.5)	0.035
Senior high school	57 (1.4)	43 (0.9)	41 (0.8)	69 (1.4)	0.903
Vocational high school	7 (1.4)	23 (2.0)	30 (2.4)	29 (2.5)	0.158
**Social exclusion, n (%)**					
Total	531 (3.1)	680 (3.6)	876 (4.4)	1158 (5.8)	< 0.001
Male	266 (3.1)	369 (3.8)	453 (4.5)	603 (5.9)	< 0.001
Female	265 (3.1)	311 (3.4)	423 (4.4)	555 (5.8)	< 0.001
Urban	295 (3.2)	354 (3.4)	527 (4.8)	542 (4.9)	< 0.001
Rural	236 (2.9)	326 (4.0)	349 (3.9)	616 (6.9)	< 0.001
Primary school	208 (3.3)	219 (3.6)	364 (5.5)	586 (8.5)	< 0.001
Junior high school	195 (3.0)	266 (4.1)	303 (4.5)	337 (4.8)	< 0.001
Senior high school	117 (2.9)	144 (2.9)	139 (2.7)	182 (3.7)	0.039
Vocational high school	11 (2.2)	51 (4.5)	70 (5.5)	53 (4.6)	0.071
**Threats, n (%)**					
Total	349 (2.0)	448 (2.4)	546 (2.8)	433 (2.2)	0.130
Male	221 (2.5)	265 (2.8)	337 (3.3)	270 (2.6)	0.341
Female	128 (1.5)	183 (2.0)	209 (2.2)	163 (1.7)	0.267
Urban	189 (2.0)	218 (2.1)	271 (2.5)	264 (2.4)	0.028
Rural	160 (2.0)	230 (2.8)	275 (3.1)	169 (1.9)	0.924
Primary school	134 (2.1)	146 (2.4)	257 (3.9)	169 (2.5)	0.009
Junior high school	133 (2.0)	201 (3.1)	187 (2.7)	163 (2.3)	0.617
Senior high school	77 (1.9)	72 (1.4)	69 (1.4)	72 (1.5)	0.104
Vocational high school	5 (1.0)	29 (2.5)	33 (2.6)	29 (2.5)	0.184
